# (Pro)renin Receptor Regulates Phosphate Homeostasis in Rats *via* Releasing Fibroblast Growth Factor-23

**DOI:** 10.3389/fphys.2022.784521

**Published:** 2022-02-11

**Authors:** Aihua Lu, Min Pu, Shiqi Mo, Jiahui Su, Jiajia Hu, Chunling Li, Weidong Wang, Tianxin Yang

**Affiliations:** ^1^Institute of Hypertension, Zhongshan School of Medicine, Sun Yat-sen University, Guangzhou, China; ^2^Department of Internal Medicine, University of Utah and Veterans Affairs Medical Center, Salt Lake City, UT, United States

**Keywords:** (pro)renin receptor, fibroblast growth factor 23, phosphate homeostasis, Na^+^-dependent Pi transporter, parathyroid hormone

## Abstract

Phosphate (Pi) is one of the basic necessities required for sustenance of life and its metabolism largely relies on excretory function of the kidney, a process chiefly under the endocrine control of bone-derived fibroblast growth factor 23 (FGF23). However, knowledge gap exists in understanding the regulatory loop responsible for eliciting phophaturic response to Pi treatment. Here, we reported a novel role of (pro)renin receptor (PRR) in mediating phosphaturic response to Pi treatment *via* upregulation of FGF23 production. Male Sprague-Dawley rats were pretreated for 5 days *via* osmotic pump-driven infusion of a PRR antagonist PRO20 or vehicle, and then treated with high Pi (HP) solution as drinking fluid for the last 24 h. PRO20 reduced HP-induced Pi excretion by 42%, accompanied by blunted upregulation of circulating FGF23 and parathyroid hormone (PTH) and downregulation of renal Na/Pi-IIa expression. In cultured osteoblast cells, exposure to HP induced a 1.56-fold increase in FGF23 expression, which was blunted by PRO20 or siRNA against PRR. Together, these results suggest that activation of PRR promotes phosphaturic response through stimulation of FGF23 production and subsequent downregulation of renal Na/Pi-IIa expression.

## Introduction

Phosphate (Pi) is an essential nutrient and component of the human body. Adequate phosphate balance is essential for the maintenance of fundamental cellular functions of the mammalian system, ranging from energy metabolism to mineral ion metabolism (Gaasbeek and Meinders, 2005). The kidney plays a pivotal role in maintenance of Pi homeostasis by adjustment of reabsorption and excretion (Shimada et al., 2004a; Urakawa et al., 2006). In the kidney, most of the filtered Pi is reabsorbed across the proximal tubule cells ([Bibr B26][Bibr B19]). Evidence from physiological studies suggests that Na^+^-dependent Pi transporters in the brush-border membrane (BBM) of proximal tubular cells mediate the rate-limiting step in the overall Pi-reabsorptive process (Murer et al., 2000, 2003). An alteration of proximal tubular reabsorption of Pi in kidney was thought to depend on the abundance of NaPi-lla (Npt2a) or NaPi-llc (Npt2c) proteins residing in the BBM (Biber and Murer, 1994; Busch et al., 1994; Shirazi-Beechey et al., 1996). Na^+^- Pi cotransporter knock out mouse demonstrated that NaPi-lla was responsible for approximately 70% of BBM Na/Pi cotransport activity (Beck et al., 1998; Murer et al., 2004).

Renal handling of Pi is tightly regulated by endocrine hormones, particularly fibroblast growth factor 23 (FGF23), vitamin D_3_, and PTH (Pfister et al., 1998; Shimada et al., 2004a; Liu et al., 2006; Farrow et al., 2009; Gattineni et al., 2009; Guo et al., 2013). Among these, FGF23 is a bone-derived hormone secreted by osteoblasts and osteocytes in response to increased Pi concentration as well as vitamin D (Saito et al., 2005; Antoniucci et al., 2006; Perwad et al., 2007). FGF23 acts on the distal convoluted tubule that may trigger a cascade that reduces proximal tubular Pi reabsorption (Farrow et al., 2009). Studies in animal models have shown that increased serum concentrations of FGF23 lead to renal Pi wasting through downregulation of Npt2a and Npt2c in the proximal tubule (PT) apical membrane (Larsson et al., 2004; Shimada et al., 2004b).

(Pro)renin receptor (PRR) is a member of the renin-angiotensin system (RAS) (Nguyen et al., 2002) and generally thought to serve as a specific receptor for both prorenin and renin. PRR is composed of a large N-terminal extracellular domain, a single transmembrane domain, and a short cytoplasmic domain (Burckle and Bader, 2006). The full length PRR (fPRR) is cleaved by site-1 protease (S1P) to generate N-terminal soluble PRR (sPRR) and the C-terminal membrane-bound M8-9 fragment (Nakagawa et al., 2017). Increasing evidence has demonstrated that PRR-mediated activation of the intrarenal RAS plays an essential role in renal handling of Na^+^ and water balance (Gonzalez and Prieto, 2015; Lu et al., 2016a,b; Quadri and Siragy, 2016; Peng et al., 2017; Prieto et al., 2017). Activation PRR triggers multiple signaling transduction pathways such as β-catenin signaling and thus can act in a RAS-independent manner (Kouchi et al., 2017; Li et al., 2017; Gao et al., 2020). So far, there is no prior research to address a potential role of PRR in regulation of Pi homeostasis. The overall goal of the present study was to test the role of PRR in phosphaturic response to HP treatment and further to address the underlying mechanism.

## Materials and Methods

### Animals

Male Sprague-Dawley rats (220–270 g) were purchased from the Medical Experimental Animal Center at Sun Yat-sen University. All animal protocols were conformed to the Experimental Animal Management Regulations of Sun Yat-sen University. Rats were acclimated in metabolic cages for 3 days prior to the start of the study. Rats were randomly divided into three experimental groups (*N* = 5 per group): (1) control group, (2) HP group, or (3) HP + PRO20 group. Animals in HP and HP + PRO20 groups drank high phosphate fluid (5 × phosphate buffered saline, pH 7.4, [Pi] = 50 mM) for 24 h (Ide et al., 2016) and the control group drank tap water. Five days prior to HP treatment, osmotic minipump (2001, Alzet, United States) was implanted to deliver vehicle or PRO20 at 700 μg/kg/d as previously described (Wang et al., 2016, 2020). Twenty four-hour urine was collected using metabolic cages.

### Plasma and Urine Parameters

Plasma and urine creatinine was determined by the QuantiChrom™ Creatinine Assay Kit (DICT-500, BioAssay Syatems, United States). Plasma and urine sodium, potassium and chlorine levels were determined by the Sodium, Potassium and Chlorine Assay Kit, respectively (Nanjing Jiancheng Bioengineering Institute, China). Plasma and urine phosphorus and calcium levels were determined by the Micro Blood Phosphorus and Calcium Concentration Assay Kit, respectively (Solarbio life sciences, China). Plasma and urine soluble PRR (sPRR) levels were determined by the ELISA kit (27782, IBL, Japan). Plasma FGF23, PTH and 1,25(OH)_2_D_3_ concentrations were assayed using the ELISA kits (Cloud-Clone Corp., China). All of these ELISA assays were performed according to the manufacturer’s protocols.

### Isolation of Renal Brush-Border Membranes

Renal BBMs were isolated by double magnesium chloride (MgCl_2_) precipitation as previously described (Gattineni et al., 2009) with minor modifications. After removal of the renal capsule, the renal cortex was isolated and homogenized in 2 ml of cold 2 × homogenization buffer (12 mM Tris pH 7.4, 300 mM mannitol, 5 mM EGTA). MgCl_2_ was added to a final concentration of 12 mM and samples were incubated on ice for 15 min with occasional mixing. Then the aggregated membranes were removed by 15-min centrifugation at 3,000 *g* and 4°C, and the supernatant was then centrifuged for 30 min at 40,000 *g* and 4°C. The pellet was resuspended in 1 ml of 1 × cold homogenization buffer supplemented with 12 mM MgCl_2_. After a second incubation and 15-min centrifugation at 3,000 *g* and 4°C and the supernatant was recovered and centrifuged at 40,000 *g*, 4°C, for 30 min. The BBM pellets were resuspended in RIPA buffer. All solutions were supplemented with protease inhibitors (1 mM PMSF).

### Immunoblotting

Protein samples were fractionated on SDS-PAGE (30 μg/well) and transferred to a nitrocellulose membrane (Millipore). Immunoblots were incubated overnight at 4°C with primary antibodies including anti-ACE (1:1,000, GTX100923, GeneTex, United States), anti-AGT (1:1,000, GTX103824, GeneTex, United States), anti-renin (1:1,000, sc-133145, Santa, United States), anti-PRR (1:1,000, HPA003156, Sigma, United States), anti-Npt2a (1:1,000, A6742, Abclonal, China), anti-Npt2c (1:1,000, ab155986, Abcam, United Kingdom) or anti-β-actin (1:10,000, A1978, Sigma, United States) antibody in 1.5% (w/v) bovine serum albumin (BSA, Sigma, United States) in a TBS-T buffer [150 mM NaCl, 10 mM Tris (pH 7.4/HCl), 0.2% (v/v) Tween-20]. After washing, membranes were incubated with horseradish peroxidase-conjugated secondary antibodies (1:3,000, Thermo Fisher Scientific™ Pierce™). Specific signal was visualized by ECL kit (Thermo Fisher Scientific™ Pierce™). The protein bands were detected using Amersham Imager 600 and quantified by Image Pro Plus version 6.0 software (Molecular Dynamics).

### Quantitative Reverse Transcriptase PCR

Total RNA was extracted using Trizol (TRIzol, Invitrogen) following manufacturer’s instructions. RNA concentrations were quantified at 260 nm, and purity and integrity were determined using NanoDrop 2000. Reverse transcription was performed using iScript™ cDNA Synthesis Kit (Bio-Rad, United States). The mRNA expression was measured by quantitative RT-PCR using SYBR^®^ *Premix Ex Taq*™ II (Tli RNaseH Plus, TaKaRa, China). The following primers were used: ACE: 5′-GAGCCATCCTTCCC–TTTTTC-3′ (forward) and 5′-CCACATGTTCCCTAGCAG-GT-3′ (reverse), AGT: 5′-AGCATCCTCCTTGAACTCCA-3′ (forward) and 5′-TGATTTT TGCCCAGGAT- -AGC-3′ (reverse), renin: 5′-GATCACCATG AAGGGG-GTCTCTGT-3′ (forward) and 5′-GTTCCTGAAG GGATTCTTTTGCAC-3′ (reverse), PRR: 5′-CTGGTGGCG- -GGTGCTTTAG-3′ (forward) and 5′-GCTACGTCTGGGAT-TC GATCT-3′ (reverse), Npt2a: 5′-GCCAGCATGACGTTTG TTGT-3′ (forward) and 5′-ATCACACCCAGG–CCAATGAG-3′ (reverse), Npt2c:5′-TGACTGTCCAAGCGT-CTGTC-3′ (forward) and 5′-TTCATCCCGATCCCCTGACT-3′ (reverse). GAPDH served as an internal control and its primer sequences were: 5′-GTCTTCACTACCA-TGGAGAAGG-3′ (forward) and 5′-TCATGGATGACCTT-GGCCAG-3′ (reverse).

### Immunohistochemistry

Under anesthesia, kidneys were harvested and fixed with 10% paraformaldehyde. Paraffin embedded kidney sections were processed for IHC as previously described (Wang et al., 2015). Primary antibody for PRR (1:200, ab40790, Abcam, United Kingdom) was incubated overnight at 4°C. Sections were washed three times with 0.01 M PBS buffer and secondary antibody horseradish peroxidase (HRP)-conjugated goat anti-rabbit (1:300, Thermo Fisher Scientific) was incubated at room temperature for an hour. The staining procedure was performed using DAB Horseradish Peroxidase Color Development Kit (P0202, Beyotime Biotechnology, China) according to the manufacturer’s protocols. Immunohistochemical staining was detected with an Olympus BX 63 microscope (20 × and 40 × objective).

### Cell Culture

The MC3T3-E1 cells were obtained from as a gift from Dr. Zhi Tan (Sun Yat-sen University). Cells were cultured in MEM-αalpha (Thermo Fisher Scientific) supplemented with 10% fetal bovine serum, and 1% penicillin-streptomycin. Cells were seeded on 6 well plates. After 24 h, the cells were starved in media containing 0.5% FBS for 24 h. Then the cells were treated with 10 mM Pi for another 24 h. To evaluate the effects of PRR on the levels of FGF23, PRO20 was given at 10 nM. To further verify the involvement of PRR, PRR was silenced by transfecting the cells with siRNA against PRR. Scrambled siRNA served as a control. SiRNA for mouse PRR and control siRNA were purchased from Ruibo Biotech (Guangzhou, China). After the treatment, the medium was collected and assayed for sPRR or FGF23 assays (EK5626, SAB, United States).

### Statistical Analysis

Data is expressed as mean ± standard error (SEM). All data points were included for analyses. Samples sizes were determined based on similar previous studies. Statistical analysis for animal and cell cultures experiments was performed by means of one-way analysis of variance (ANOVA) for multiple-group comparison or Student’s *t*-test for two-group comparison. A *p*-value below 0.05 was considered statistically significant.

## Results

### Activation of (Pro)renin Receptor and Other Renin-Angiotensin System Components by High Pi Intake

**FIGURE 1:**
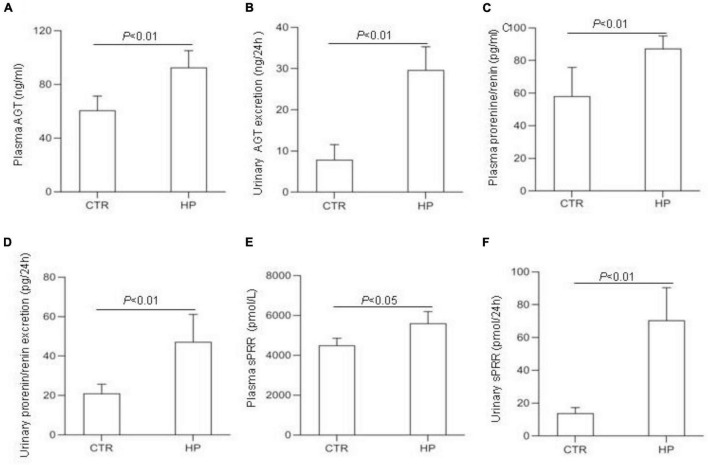
Analysis of the RAS components in rat plasma and urine following HP intake. **(A)** Plasma angiotensin (AGT) concentration. **(B)** Urinary AGT excretion. **(C)** Plasma renin concentration. **(D)** Urinary renin excretion. **(E)** Plasma soluble (pro)renin receptor (sPRR) concentration. **(F)** Urinary sPRR excretion. CTR, control; HP, high Pi intake *N* = 5 per group. Data are Mean ± SEM.

**FIGURE 2:**
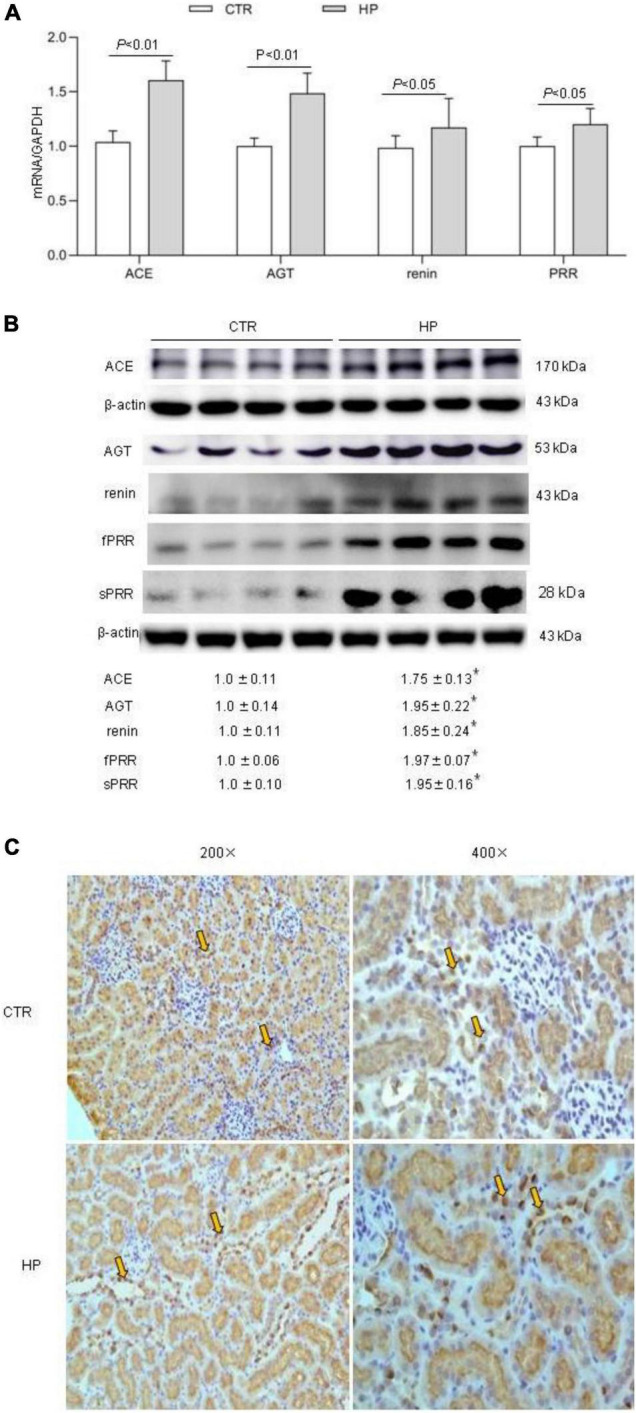
Analysis of the RAS components in rat kidneys following HP intake. **(A)** qRT-PCR detection of renal transcripts of ACE, AGT, renin, and PRR. GAPDH was used as internal reference. **(B)** Immunoblotting analysis of renal expression of ACE, AGT, renin, fPRR and sPRR. The values indicate the corresponding densitometry analysis. β-actin was used as an internal reference. **(C)** Representative images of PRR immunostaining. Arrows indicate positive staining (200×, 400×). *N* = 5 per group. Data are Mean ± SEM. *p < 0.05 vs. CTR.

To test whether HP activated the RAS, we determined the levels of RAS components in urine and plasma from rats on normal Pi (NP) or HP intake using ELISA. The results showed that the levels angiotensinogen (AGT), renin, sPRR in urine and plasma from the HP group were significantly increased as compared with NP controls (Figure 1). By qRT-PCR, renal cortical mRNA expression of angiotensin-converting enzyme (ACE), AGT, renin, PRR were all increased in the HP group as compared with NP controls (Figure 2A). These results have been validated by Western blotting analysis. Of note, this analysis detected increases in the protein abundances of both PRR and sPRR in the kidney of HP rats (Figure 2B). By immunohistochemistry, PRR protein expression was elevated in the collecting duct by HP treatment (Figure 2C), a pattern consistent with intercalated cell labeling as reported previously (Wang et al., 2016).

### Effect of PRO20 on Phosphaturic Response to High Pi Intake

SD rats drank tap water, HP fluid alone or in combination with PRO20 treatment. Basic physiological data is shown in Table 1. Fluid intake was less but urine output was higher in HP rats as compared with vehicle control. This was paralleled with increased 24-h urinary excretion of Na^+^, K^+^, and Cl^–^ induced by HP treatment. However, plasma creatinine and osmolality remained unchanged. None of these parameters were affected by PRO20.

**TABLE 1:** General physiological data in rats.

	CTR	HP	HP + PRO20
Fluid intake (ml/24 h)	25.42 ± 0.63	12.61 ± 0.38**	12.21 ± 0.58**
Urine volumes (ml/24 h)	10.41 ± 1.20	14.21 ± 0.48*	14.01 ± 0.31*
Plasma creatinine (mg/dl)	0.58 ± 0.01	0.58 ± 0.01	0.57 ± 0.02
Plasma-Na^+^ (mmol/l)	126.5 ± 1.38	126.12 ± 1.73	125.47 ± 2.31
Plasma-K^+^ (mmol/l)	3.68 ± 0.06	3.65 ± 0.07	3.67 ± 0.14
Plasma-Cl^–^ (mmol/l)	113.74 ± 1.97	115.04 ± 0.84	111.84 ± 1.55
Urinary creatinine (mg/24 h)	7.18 ± 0.43	8.07 ± 0.15	7.80 ± 0.19
Urinary Na^+^ (mmol/24 h)	1.03 ± 0.03	4.58 ± 0.18**	4.41 ± 0.27**
Urinary K^+^ (mmol/24 h)	3.43 ± 0.18	3.50 ± 0.09	3.46 ± 0.12
Urinary Cl^–^ (mmol/24 h)	1.72 ± 0.10	3.31 ± 0.05**	3.27 ± 0.08**
Plasma osmolarity (mosn/kg⋅H_2_O)	312 ± 2.06	313 ± 0.94	312 ± 1.27
Urine osmolarity (mosn/kg⋅H_2_O)	1,340.4 ± 34.60	1,407.00 ± 45.52	1,379.50 ± 49.75

Data represent the means ± SEM. *p < 0.05, **p < 0.01 vs. CTR. CTR, control.

**FIGURE 3:**
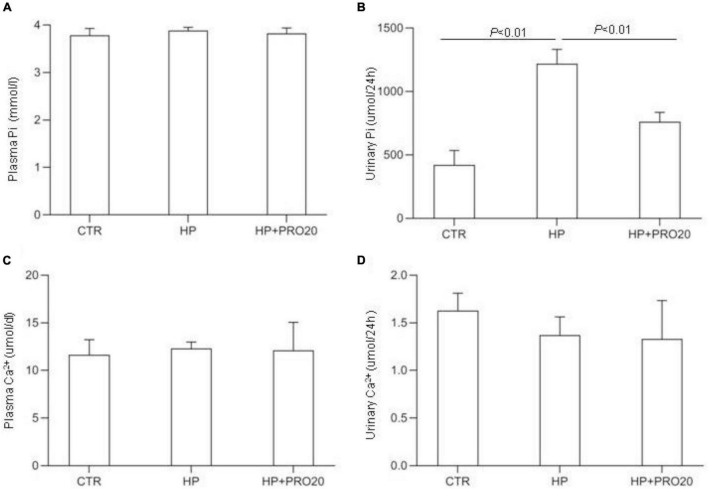
Serum and urine biochemical parameters in NP, HP and HP + PRO20 rats. **(A)** Plasma Pi concentration; **(B)** 24-h urinary Pi excretion; **(C)** plasma Ca^2+^ concentration; **(D)** 24-h urinary Ca^2+^ excretion. *N* = 5 per group. Data are Mean ± SEM.

**FIGURE 4:**
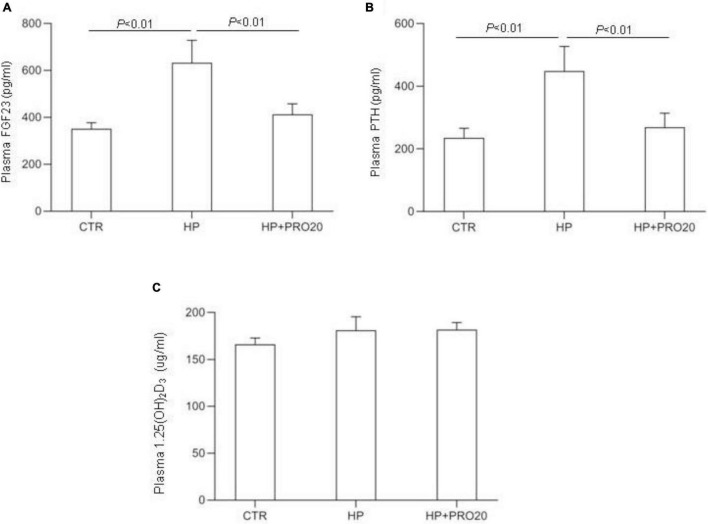
Levels of plasma FGF23 **(A)**, plasma PTH **(B)**, and plasma 1,25(OH)_2_D_3_ **(C)** were measured in CTR, HP, and HP + PRO20 rats. FGF23, fibroblast growth factor 23; PTH, parathyroid hormone; *N* = 5 per group. Data are Mean ± SEM.

To address the functional role of PRR in Pi homeostasis, we examined the effect of PRO20 on phosphaturic response to HP intake. HP intake induced a significant increase in urinary Pi excretion within 24 h and this increase was blunted by PRO20 treatment (Figure 3B). In parallel, HP intake elevated circulating FGF23 and PTH, both of which were nearly normalized by PRO20 treatment (Figures 4A,B). Despite reduced urinary Pi excretion, PRO20 treatment in HP rats did not affect plasma Pi concentration (Figure 3A). In a sharp contrast, plasma Ca^2+^ concentration (Figure 3C), urinary Ca^2+^ excretion (Figure 3D), or plasma 1,25(OH)_2_D_3_ (Figure 4C) were unaffected by HP intake or PRO20 treatment.

In a separate experiment, we examined the effect of PRO20 on several key parameters of Pi homeostasis in 7-wk-old male SD rats under basal condition (*n* = 5 per group). The data showed that PRO20 had no effect on urinary Pi excretion (PRO20 429.2 ± 16.8 vs. CTR 432.4 ± 17.8 μmol/24 h, *p* > 0.05), plasma Pi concentration (PRO20 2.89 ± 0.06 vs. CTR 2.90 ± 0.08, mmol/L, *p* > 0.05), plasma FGF-23 (PRO20 374.3 ± 15.4 vs. CTR 381.3 ± 10.2 pg/ml, *p* > 0.05), or urine volume (PRO20 9.75 ± 0.42 vs. CTR, 10.45 ± 0.85 ml, *p* > 0.05).

**FIGURE 5:**
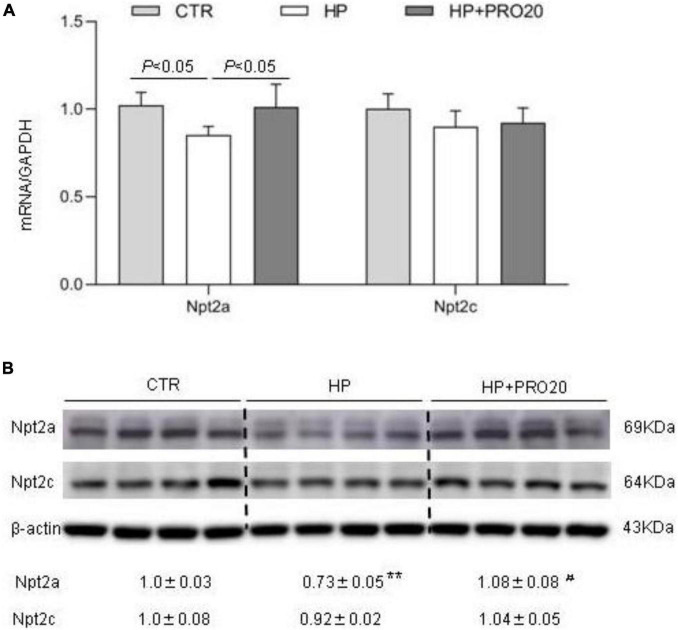
Analysis of renal expression of Pi transporters in NP, HP and HP + PRO20 rats. **(A)** qRT-PCR analysis of renal mRNA expression of Npt2a and Npt2c. GAPDH was used as internal reference. **(B)** Immunoblotting analysis of Npt2a, and Npt2c protein expression. Brush border membrane was isolated from the kidney of all groups. The values indicate the corresponding densitometry analysis. β-actin was used as an internal reference. *N* = 5 per group. Data are Mean ± SEM. **p < 0.01 vs. CTR, ^#^*p* < 0.05 vs. HP.

Downregulation of renal expression of sodium-phosphate cotransporters is a key determinant of phosphaturic response during HP intake (Murer et al., 1999; Hernando et al., 2001; Giral et al., 2009; Bourgeois et al., 2013; Forster et al., 2013; Zhuo et al., 2020). Therefore, we determined renal expression of Npt2a and Npt2c by both qRT-PCR and Western blotting analysis. In response to HP intake, renal cortical mRNA expression of Npt2a was significantly decreased, which was blunted by PRO20 treatment (Figure 5A). In contrast, the mRNA expression of Npt2c showed no significant changes in the three groups (Figure 5A). Meanwhile, we examined the abundance of sodium-phosphate cotransporters in the kidney BBM by Western blotting analysis. The protein abundance of Npt2a in BBM was downregulated by HP intake as compared with the NP control and this downregulation was prevented by PRO20 (Figure 5B). In contrast, no change was observed in protein abundance of Npt2c in BBM (Figure 5B).

### Effect of (Pro)renin Receptor on FGF23 Production in Cultured MC3T3-E1 Cells

**FIGURE 6:**
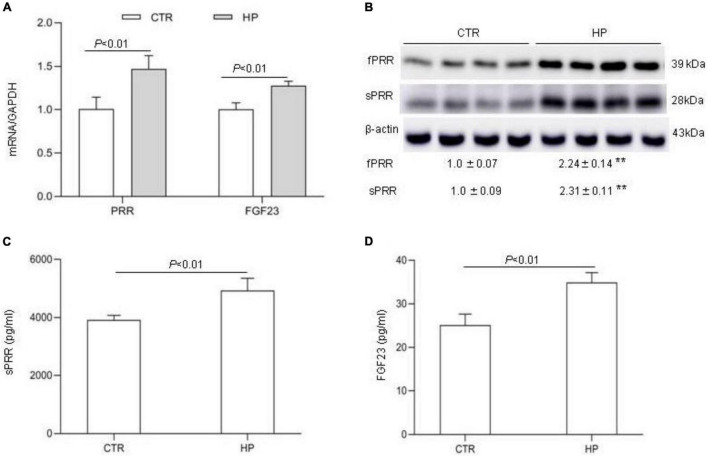
PRR mediation of FGF23 production in cultured osteoblast cells in response to Pi treatment. MC3T3-E1 cells were exposed to vehicle or 10 mM Pi for 24 h, followed by analysis of the levels of FGF23 and PRR/sPRR. **(A)** qRT-PCR analysis of mRNA expression of PRR and FGF23. GAPDH was used as internal reference. **(B)** Immunoblotting analysis of protein abundances of fPRR and sPRR. The values indicate the corresponding densitometry analysis. β-actin was used as an internal reference. **(C)** ELISA detection of medium sPRR. **(D)** ELISA detection of medium FGF23. *N* = 4, Data are Mean ± SEM. **p < 0.01 vs. CTR.

The observation of suppressed circulating FGF23 concentration by PRO20 treatment during HP intake prompted us to speculate that the bone might be a potential site of PRR regulation of FGF23 release. To address this possibility, we conducted *in vitro* experiments using MC3T3-E1 cells, a mouse osteoblast cell line. The cells were exposed to normal or high Pi (10 mM Pi) for 24 h followed by examination of expression of FGF23 as well as PRR. qRT-PCR results showed that the expression of PRR and FGF23 mRNA was both significantly increased by HP treatment (Figure 6A). Consistent with this result, Western blotting analysis demonstrated significant elevations of protein abundance of full-length PRR (fPRR) and sPRR (Figure 6B). ELISA results showed that the concentrations of sPRR and FGF23 in the medium were significantly increased by HP treatment (Figures 6C,D).

**FIGURE 7:**
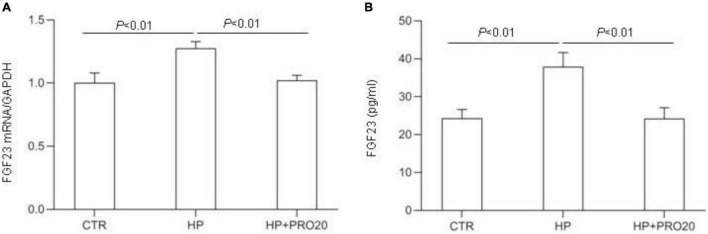
Effects of PRO20 on the expression of FGF23 in MC3T3-E1 cells. The MC3T3-E1 cells were pretreated with PRO20 (10 μM) for 1 h, then treated with Pi (10 mM) for 24 h. **(A)** qRT-PCR analysis of mRNA expression of FGF23. GAPDH was used as internal reference. **(B)** ELISA detection of medium FGF23. *N* = 4, Data are Mean ± SEM.

Next, we examined the functional role of PRR in regulation of the production of FGF23 in the MC3T3-E1 cells by using PRO20. The cells were pretreated for 1 h with 10 μM PRO20 and then treated with 10 mM Pi for 24 h. By qRT-PCR, HP treatment increased the expression of FGF23 mRNA, and this increase was blunted by PRO20 (Figure 7A). This result was subsequently validated at protein level by ELISA (Figure 7B).

**FIGURE 8:**
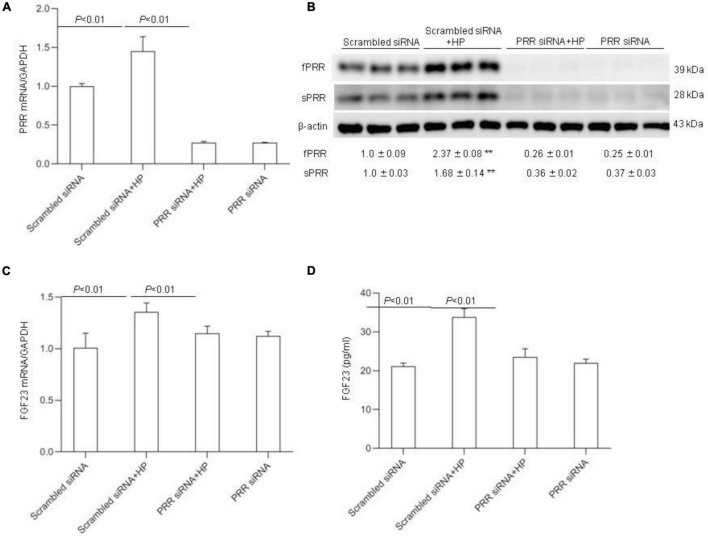
Effects PRR siRNA on HP induced FGF23 levels in MC3T3-E1 cells. The cells were transfected with scrambled or siRNA against PRR, and then treated with vehicle or 10 mM Pi for 24 h. **(A)** qRT-PCR detection of PRR mRNA expression normalized by GAPDH. **(B)** Immunoblotting analysis of protein abundances of fPRR and sPRR were detected by Western blotting. The values indicate the corresponding densitometry analysis. β-actin was used as an internal reference. **(C)** qRT-PCR analysis of mRNA expression of FGF23 normalized by GAPDH. **(D)** ELISA analysis of medium FGF23. *N* = 4, Data are Mean ± SEM, **p < 0.01 vs. CTR.

To further verify the above-mentioned results obtained with the pharmacological approach, we conducted independent experiments using siRNA approach to knockdown PRR. The efficacy of the gene knockdown was confirmed by qRT-PCR and Western blotting analysis (Figures 8A,B). PRR knockdown significantly blocked HP-induced FGF23 expression as assessed by qRT-PCR (Figure 8C) and ELISA (Figure 8D).

## Discussion

PPR is a multi-functional protein critically involved in renal handling of Na^+^, K^+^ and water through RAS-dependent or -independent mechanisms (Lu et al., 2016a,b; Quadri and Siragy, 2016; Peng et al., 2017; Prieto et al., 2017; Xu et al., 2017; Ramkumar et al., 2018; Fu et al., 2019). The present study explored phosphaturic role of PRR during 24-h Pi loading. Following HP intake, the levels of circulating sPRR along with renal expression of PRR and other components of the RAS were all elevated in parallel with increased plasma FGF23 and PTH. PRR antagonism with PRO20 effectively suppressed HP-induced FGF23 and PTH levels and urinary Pi excretion albeit with unchanged plasma Pi concentration. Cell culture experiments offered a cellular mechanism of PRR regulation of FGF23 expression in an osteoblast cell line.

In response to HP intake, the levels of PRR/sPRR were elevated as evidenced by increased circulating sPRR, the cleavage product of PRR, and renal expression of PRR. The source of sPRR under HP intake remains obscure. Immunostaining mapped HP-induced PRR expression in the collecting duct (CD) with a pattern of labeling in intercalated cells (ICs) as previously reported (Wang et al., 2016). It is intriguing that the CD may serve as a potential site for the generation of sPRR during HP intake although other organs such as bone or parathyroid gland may also be involved. ICs were initially thought to primarily regulate acid-base metabolism. However, emerging evidence suggests novel sensing function of ICs during urinary tract infection and acute kidney injury (Miao and Abraham, 2014; Azroyan et al., 2015; Battistone et al., 2020). More recent evidence suggests a paracrine mechanism whereby mediators such as sPRR or prostaglandins are produced by ICs and act in the neighboring principal cells of the CD to regulate Na^+^ and water reabsorption in the distal nephron (Lu et al., 2016a,b; Xu et al., 2020). Our results indicate a possibility that IC PRR may be involved in regulation of Pi homeostasis by releasing sPRR that may target other organs to control production of phosphaturic hormones such as FGF23. The involvement of IC-derived sPRR in renal handling of Pi should be tested by future investigation.

Although PRR was initially identified as a specific receptor for prorenin and renin, its relationship with RAS has been debated (Binger and Muller, 2013). Recently, abundant evidence from our group strongly supports PRR as an important regulator of intrarenal RAS during water deprivation (Wang et al., 2016), angiotensin II-induced hypertension (Wang et al., 2014, 2017) and chronic kidney injury (Fang et al., 2018), favoring PRR as integrative member of the RAS. Along this line, the present study offered new evidence of activation of the RAS during HP intake. In this regard, HP treatment induced plasma and urinary excretion and renal expression of AGT and renin in parallel with elevated levels of PRR/sPRR. Future studies are needed to determine dependence of the canonical RAS components on PRR and its functional contribution to Pi homeostasis.

We employed a pharmacological approach to provide functional evidence for a novel role of PRR in mediating phosphaturic response to HP intake in rats. PRO20 has been extensively characterized as a highly specific and effective inhibitor of PRR owing to its peptide decoy activity (Danser, 2015; Li et al., 2015). Administration of PRO20 was highly effective in attenuating HP-induced urinary Pi excretion and phosphaturic hormones such as FGF23 and PTH. These hormones primarily target the kidney to downregulate abundance of Npt2a in the brush border of proximal tubules. Indeed, HP-induced downregulation of Npt2a was prevented by PRO20 treatment. The result support phosphaturic role of PRR during Pi treatment. Of note, despite impaired phosphaturic response, PRO20 didn’t elevate plasma Pi concentration during HP treatment. This might be due to the relatively short duration of HP treatment. Under this condition, effective compensatory mechanism might be activated and able to maintain normal level of plasma Pi concentration.

Osteoblast cells are a known source of FGF23 production during HP intake (Liu et al., 2003; Ferrari et al., 2005; Perwad et al., 2005; Lindberg et al., 2015; Goltzman et al., 2018). Considering the observation that PRO20 effectively suppressed HP-induced circulating level of FGF23, we hypothesized that FGF23 production might be under the direct control of PRR in cultured osteoblast cells. Using a cell culture model of osteoblast cells, we obtained compelling evidence that HP-induced FGF23 mRNA expression and release were blunted by PRO20 and siRNA against PRR. We provided further evidence that PRR expression was stimulated by HP treatment. An issue may arise that the relative importance of PRR in osteoblast cells vs. the kidney for the control of FGF23 production remains unclear and should warrant future investigation.

Besides FGF23, PTH is another important regulator of Pi metabolism (Graciolli et al., 2009; Lombardi et al., 2020). In the present study, we were able to show an inhibitory effect of PRO20 on HP-induced plasma PTH level, indicating a potential role of PRR in regulation of the release of PTH, presumably from parathyroid gland. There is no information about expression and function of PRR in this organ in the context of PTH regulation. We would like to acknowledge this major limitation of the present study.

We would also like to acknowledge the limitation of the HP protocol used in the present study although this protocol has been validated by a previous study (Ide et al., 2016). The HP fluid contains high NaCl which may confoundingly influence Pi transport in the kidney through modulation of status of NaPi transporter. This possibility is suggested by the previous observation that subcellular distribution of NaPi-2 was altered following high salt diet (Yang et al., 2008), but with unchanged total abundance of this transporter. In contrast, as shown by the present study, the total abundance of NaPi-2 was downregulated by 24-h HP intake. This result seems to support a primary role of NaPi-2 in regulating homeostasis of Pi, probably not Na^+^. Indeed, besides NaPi-2, Na^+^ transport occurs *via* numerous other Na^+^ transporters and channels in various nephron segments. Additionally, it seems hard to explain why HP intake reduced fluid intake that was contradictorily associated with increased urine output and urinary Na^+^ excretion. Fortunately, we found no sign of severe dehydration as evidenced by unchanged plasma osmolality. This might be due to the short duration of the experiment and fluid balance can be maintained by activation of compensatory mechanisms.

**FIGURE 9:**
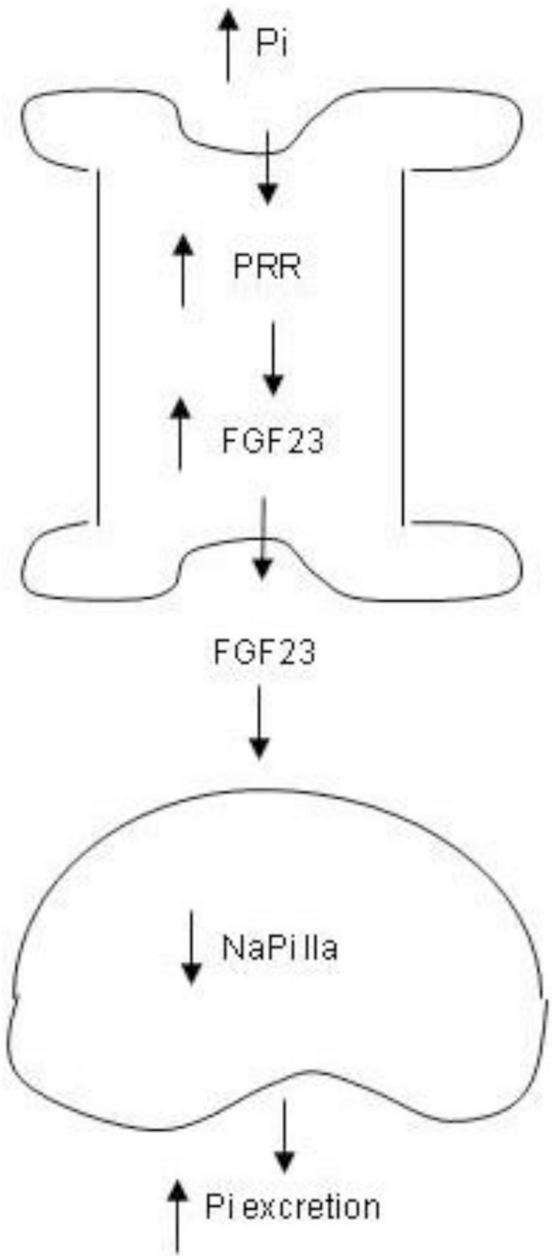
Schematic illustration of the PRR-mediated phosphaturic response. In response to high Pi, PRR expression is elevated in the bone, leading to elevation of FGF23 production. Then FGF23 is released to the circulation and acts in the kidney to suppress NaPi IIa expression, resulting in increased Pi excretion.

In conclusion, we for the first time identified PRR as a novel mediator of phosphaturic response to HP intake. The phosphaturic action of PRR seemed to be mediated by stimulation of production of FGF23 as well as PTH (Figure 9). *In vitro* evidence from cultured osteoblast cells demonstrated that PRR directly mediated HP-induced FGF23 release. Overall, the present study has uncovered a previously undescribed PRR/FGF23 axis in regulation of Pi homeostasis.

## Data Availability Statement

The raw data supporting the conclusions of this article will be made available by the authors, without undue reservation.

## Ethics Statement

The animal study was reviewed and approved by the Institutional Animal Care and Use Committee, Sun Yat-sen University.

## Author Contributions

TY and AL designed the research and wrote the manuscript. AL, JS, SM, MP, and JH performed the experiments. AL analyzed the data. AL, CL, WW, and TY edited and revised manuscript. TY approved final version of manuscript. All authors approved the final version of the manuscript.

## Conflict of Interest

The authors declare that the research was conducted in the absence of any commercial or financial relationships that could be construed as a potential conflict of interest.

## Publisher’s Note

All claims expressed in this article are solely those of the authors and do not necessarily represent those of their affiliated organizations, or those of the publisher, the editors and the reviewers. Any product that may be evaluated in this article, or claim that may be made by its manufacturer, is not guaranteed or endorsed by the publisher.
